# Genome Sequence of *Mycobacterium* Phage Waterfoul

**DOI:** 10.1128/genomeA.01281-16

**Published:** 2016-11-17

**Authors:** Paige N. Jackson, Ella K. Embry, Christa O. Johnson, Tiara L. Watson, Sayre K. Weast, Caroline J. DeGraw, Jessica R. Douglas, J. Michael Sellers, William A. D’Angelo, Dmitri V. Mavrodi

**Affiliations:** a Department of Biological Sciences, University of Southern Mississippi, Hattiesburg, Mississippi, USA; b School of Polymers and High Performance Materials, University of Southern Mississippi, Hattiesburg, Mississippi, USA

## Abstract

Waterfoul is a newly isolated temperate siphovirus of *Mycobacterium smegmatis* mc^2^155. It was identified as a member of the K5 cluster of *Mycobacterium* phages and has a 61,248-bp genome with 95 predicted genes.

## GENOME ANNOUNCEMENT

Mycobacteriophage Waterfoul was isolated from soil collected in Hattiesburg, Mississippi, USA, using *Mycobacterium smegmatis* mc^2^155 as a host. The soil sample was harvested on the shoreline of a pond at the Hattiesburg Zoo (GPS coordinates 31.323056 N, 89.316389 W). The isolation and genome analysis of Waterfoul were conducted as part of the undergraduate laboratory course called “Phage Hunters,” which was organized at the University of Southern Mississippi with the help of the Science Education Alliance and the Howard Hughes Medical Institute (1).

The phage was isolated using a standard enrichment technique with incubation at 22°C. Electron microscopy revealed that Waterfoul has an isometric head of 60 nm and a flexible 200-nm-long noncontractile tail. The phage was purified and amplified, and its DNA was isolated and sequenced at the Pittsburgh Bacteriophage Institute using an Illumina MiSeq instrument. Single-end run reads were assembled to give a single contig with 3,046-fold coverage. The 61,248-bp genome is flanked by *cos* ends with 11-bp 3′ extensions of the sequence 5′-CTCAGTGGCAT. The G+C content of Waterfoul is 64.9% and is close to that of *M. smegmatis*.

Putative genes in the Waterfoul genome were identified using Glimmer (2), GeneMark (3), Aragorn (4), and tRNAscan-SE (5), followed by manual inspection and annotation revision using DNA Master (http://cobamide2.bio.pitt.edu). Gene functions were assigned using BLAST (6) and HHPred (7). A total of 94 protein-coding genes were predicted, accounting for a 93.1% coding capacity of the genome. BLASTn analysis revealed that Waterfoul is a member of the K5 subcluster and closely related to mycobacteriophages Gengar, Leston, and OkiRoe (8).

The Waterfoul genome has features common to other siphoviruses, including structural genes that encode a major capsid subunit (gp11), a maturation protease (gp8), a scaffolding protein (gp10), a terminase (gp5 and gp6), a portal protein (gp7), head-to-tail connector proteins (gp12 to gp15), a tape-measure protein (gp19), and a major (gp16) and several minor (gp20 to gp25) tail proteins. Genes involved in DNA replication and nucleic acid metabolism encoded a DNA primase/polymerase (gp65), a DNA helicase (gp66), a DnaQ-like exonuclease (gp54), an RNA ligase (gp78), a RusA resolvase (gp67), a glutaredoxin (gp62), and proteins with predicted HNH endonuclease motifs (gp63, gp64, and gp95). Regulatory proteins included an immunity repressor with the helix-turn-helix DNA binding motif (gp42) and a putative WhiB-like transcriptional regulator (gp53). Like some other temperate mycobacteriophages, Waterfoul had a tyrosine recombinase type integrase (gp39), a tRNA-Trp (CCA) (gp4), as well as the lysis system represented by the holin (gp31), lysin A (gp29), and lysin B (gp30).

### Accession number(s).

The genome of bacteriophage Waterfoul was deposited in GenBank under the accession number KX585251.
